# Short-Term Clinical Outcomes of Ready-Made Soft Splints Versus Digitally Fabricated, Customized Hard Stabilization Splints in Temporomandibular Disorders: An Exploratory Randomized Study

**DOI:** 10.3390/biomedicines14102299

**Published:** 2026-10-10

**Authors:** Sang-Yoon Park, Soo-Hwan Byun, Sae-Hoon Baek, Sung-Ah Che, Seoung-Won Cho, In-Young Park, Byoung-Eun Yang

**Affiliations:** 1 Department of Oral and Maxillofacial Surgery, Hallym University Sacred Heart Hospital, Anyang 14066, Republic of Korea; psypjy0112@naver.com (S.-Y.P.); purheit@daum.net (S.-H.B.); intse@naver.com (S.-H.B.); sungche1219@gmail.com (S.-A.C.); 2 Department of Artificial Intelligence and Robotics in Dentistry, Graduate School of Clinical Dentistry, Hallym University, Chuncheon 24252, Republic of Korea; kotneicho@gmail.com (S.-W.C.); park.iy2875@gmail.com (I.-Y.P.); 3 Institute of Clinical Dentistry, Hallym University, Chuncheon 24252, Republic of Korea; 4 Dental Artificial Intelligence and Robotics R&D Center, Hallym University Medical Center, Anyang 14066, Republic of Korea; 5 Department of Orthodontics, Hallym University Sacred Heart Hospital, Anyang 14066, Republic of Korea

**Keywords:** temporomandibular disorders, occlusal splint, stabilization splint, soft splint, 3D printing, digital workflow, modified Helkimo index, exploratory randomized study

## Abstract

**Background/Objectives**: Occlusal splints are widely used for temporomandibular disorders (TMDs), but direct evidence comparing ready-made soft splints with customized hard stabilization splints remains limited. This exploratory randomized study compared the short-term clinical outcomes of a ready-made soft splint and a digitally fabricated, customized 3D-printed hard stabilization splint, representing two distinct fabrication and delivery workflows. **Methods**: Seventy adults with TMDs were randomized 1:1 to a customized hard splint fabricated using intraoral scanning and digital light processing 3D printing or to a ready-made soft splint (n = 35 per group); 68 completed follow-up. Modified Helkimo index scores were assessed at baseline (T0), 4 weeks (T1), and 12 weeks (T2). **Results**: Scores decreased significantly from baseline in both groups at T1 and T2 (all *p* < 0.001). No statistically significant between-group difference was detected in change from baseline to T1 (Hodges–Lehmann median difference, hard minus soft = 0.00; 95% CI, −1.00 to 1.00; *p* = 0.573) or T2 (−1.00; 95% CI, −2.00 to 0.00; *p* = 0.209). **Conclusions**: No statistically significant difference in short-term clinical improvement was detected between the two splint groups. This finding should not be interpreted as evidence of equivalence or non-inferiority. Ready-made soft splints may have a limited, hypothesis-generating role as a short-term conservative or bridging option for selected patients, but adequately powered, prospectively registered non-inferiority trials are needed to define their clinical role. Trial Registration: CRIS KCT0012318 (retrospectively registered).

## 1. Introduction

Temporomandibular disorders (TMDs) comprise a heterogeneous group of musculoskeletal and neuromuscular conditions involving the temporomandibular joint (TMJ), masticatory muscles, and related craniofacial structures [1]. Patients typically present with joint pain, masticatory muscle pain, joint sounds, or limitation of mandibular movement. Because TMDs encompass both myogenous and arthrogenous conditions, accurate diagnosis is essential before selecting a treatment strategy [2]. Conservative and reversible management is generally recommended as the initial approach, including patient education, behavioral modification, pharmacologic therapy, physical therapy, and occlusal appliance therapy [3,4,5,6,7,8,9].

Occlusal splints remain among the most widely used non-invasive treatment modalities for TMDs [3,10]. Their therapeutic rationale is multifactorial: stabilization-type appliances may reduce deflective occlusal contacts, distribute occlusal loading, reduce parafunctional loading, and promote neuromuscular relaxation of the masticatory system [10,11]. Hard stabilization splints fabricated from acrylic resin have traditionally been the standard of care, offering stable occlusal contacts and durable retention, particularly when fabricated using digital workflows such as intraoral scanning and three-dimensional printing [12]. This digital light processing (DLP)-based 3D printing approach, combined with biocompatible photopolymer resins, allows for increased geometric precision and reproducibility compared with conventional analog fabrication techniques, and has recently been applied specifically to occlusal splint therapy for sleep bruxism using the same Asiga DLP printer platform employed in the present study [13]. Unlike the previous application [13] focused on fabrication methods for bruxism-specific appliances, this study assesses a wider TMD population based on DC/TMD criteria. It directly compares clinical outcomes between a digitally produced hard splint and a ready-made soft splint, using the validated modified Helkimo index within a randomized trial. Such digitally fabricated splints have been reported to improve occlusal balance and reduce joint pain in patients with TMDs [14]. However, the clinical effectiveness of splint therapy may depend on the underlying diagnosis, appliance design, fit, patient adherence, and contributing behavioral or psychosocial factors [2,5]. Customized hard splints require scanning, laboratory fabrication, and chairside adjustment before delivery.

In contrast, ready-made soft splints and semi-finished or prefabricated appliances can be delivered and adjusted chairside at the initial visit, which may benefit patients requiring immediate symptom management or in settings where cost, access, or treatment timing are limiting factors [15,16,17,18,19]. Several comparative studies support their short-term clinical utility, and some have reported outcomes comparable to those of hard splints [15,20], while others have found soft splints effective specifically for masticatory muscle pain [16].

However, other studies have raised concerns regarding the fit, retention, occlusal stability, and durability of ready-made appliances relative to customized hard splints [17,18], and have reported differences in the speed or magnitude of symptom improvement favoring hard splints in certain contexts [10]. These studies are further limited by heterogeneous diagnostic criteria, variable appliance designs, small sample sizes, short follow-up periods, and insufficient blinding of outcome assessment [10]. The clinical question of whether a readily available soft splint can provide short-term symptom improvement comparable to that of a customized hard stabilization splint therefore remains incompletely addressed. A recent systematic review found that the certainty of evidence supporting the effects of stabilization splints on TMD symptoms was very low for all assessed outcomes [21]. Several specific clinical situations further illustrate the practical value of ready-made appliances. In patients undergoing active orthodontic treatment, the dental arches are in continuous change, making customized hard splints impractical as the appliance quickly becomes ill-fitting. Patients presenting with significant mouth-opening restriction, a finding encountered in certain TMD subtypes such as disc displacement without reduction, may not tolerate conventional intraoral impression-taking, which delays the fabrication of a customized device. Similarly, in growing patients whose jaw dimensions are still developing, customized splints may require frequent replacement as the dentition changes, adding practical and financial burden to care [22,23]. In such settings, a ready-made, chairside-adjustable appliance could theoretically offer practical advantages. However, these populations were not evaluated in the present study, and the present findings should not be interpreted as supporting the use of ready-made soft splints in these specific clinical situations.

The modified Helkimo index provides a clinically applicable and validated method for repeated assessment of TMD-related dysfunction severity based on five measurable clinical components [24,25]. In a recent direct comparison against the Diagnostic Criteria for Temporomandibular Disorders (DC/TMD), the modified Helkimo index demonstrated 86% sensitivity and 84% specificity while requiring substantially less examination time (5–10 min vs. 16–20 min) [24], and independent psychometric evaluation has reported excellent inter-observer agreement and strong diagnostic accuracy (area under the curve = 0.90) [26]. The index continues to be applied in contemporary TMD research [27]. Although it does not replace multiaxial frameworks such as DC/TMD [2], its brevity, established reliability, and suitability for repeated longitudinal assessment support its use as the primary outcome instrument in the present exploratory trial.

This exploratory randomized study aimed to compare the short-term clinical outcomes of a ready-made soft splint and a customized 3D-printed hard splint in patients with TMDs using the modified Helkimo index at baseline, 4 weeks, and 12 weeks, comparing two appliances that differ not only in material but across their entire clinical workflow, from digital scanning and laboratory fabrication to chairside delivery. We hypothesized that both splint types would be associated with significant symptom improvement over time, and that the magnitude of short-term improvement would not differ significantly between groups.

## 2. Materials and Methods

### 2.1. Patients

Eligible participants were adult patients aged 18 or older with a clinical TMD diagnosis. The study used the DC/TMD Axis I criteria, supplemented by radiographic exams when needed, to confirm TMD presence [2]. A detailed, patient-level classification of DC/TMD Axis I subtypes (such as myalgia, arthralgia, disc displacement with or without reduction, or degenerative joint disease) was not systematically recorded or retained as part of the study data. Additionally, DC/TMD Axis II psychosocial assessments were not conducted. Eligible patients mainly exhibited symptoms such as masticatory muscle pain, TMJ pain, TMJ sounds, limited mandibular movement, or disc displacement-related issues, with many patients showing multiple symptoms simultaneously. The treating clinicians also used broad descriptive labels of predominantly myogenous or predominantly arthrogenous presentation in routine clinical assessment; however, these labels were not based on a pre-specified classification algorithm and were not used as formal diagnostic subtypes in the analysis. Patients were recruited from Hallym University Sacred Heart Hospital and Balance dental hospital after obtaining institutional review board approval at each participating site. The exclusion criteria were as follows: previous TMD-related splint therapy; odontogenic pain; history of trauma within the previous 6 months; more than five missing teeth; use of partial or complete dentures; abnormal condylar morphology on radiographic examination; systemic joint disease; inability or unwillingness to comply with follow-up; or refusal to participate in the study. A total of 70 patients were enrolled and randomly allocated in a 1:1 ratio to the customized hard splint group (n = 35) or the ready-made soft splint group (n = 35). All patients received standardized counseling regarding the nature of TMDs, avoidance of excessive parafunctional activity, and instructions for splint use prior to appliance delivery. Patients or members of the public were not involved in the design, conduct, reporting, or dissemination plans of this study.

### 2.2. Sample Size

The required sample size was calculated using G*Power software (version 3.1.9.7; Heinrich-Heine-Universität Düsseldorf, Düsseldorf, Germany) [28] for a two-tailed comparison between two independent groups, with a significance level of α = 0.05, a statistical power of 1 − β = 0.80, and an expected effect size of Cohen’s d = 0.75. The selection of this effect size was informed by a review of available comparative studies of soft, hard, semi-finished, or prefabricated occlusal appliances [15,17,18,20]. Among these, Poorna et al. [20] directly employed the modified Helkimo index as the outcome measure in a similar randomized comparison and reported a statistically significant between-group difference in score change at one month (*p* = 0.005), with no significant difference at three months (*p* = 0.718). The remaining referenced studies used different outcome measures and did not report Cohen’s d values directly. In the absence of a universally accepted effect size estimate derived from studies using an identical outcome measure and follow-up design, a Cohen’s d of 0.75 was selected as a conservative, moderate-to-large estimate, considered clinically meaningful, for the between-group comparison of short-term TMD symptom improvement. Under these assumptions, the minimum required sample size was 29 patients per group. This calculation was based on a conventional superiority framework rather than a pre-specified non-inferiority margin. As this exploratory study was not designed as a formal non-inferiority or equivalence trial, the sample size and statistical power described here should be interpreted as supporting detection of a between-group difference in the specified magnitude, rather than as evidence for a pre-planned equivalence assessment; this distinction is further addressed in Section 4. Accounting for possible dropout during follow-up, the target sample size was set at 35 patients per group. No interim analyses or stopping guidelines were planned or conducted, consistent with this study’s fixed-sample, single-analysis design.

### 2.3. Randomization

Patients were randomized in a 1:1 ratio to either the customized hard splint group or the ready-made soft splint group using a computer-generated simple (unrestricted) randomization sequence, without blocking or stratification, prepared in R software (version 4.3.1; R Foundation for Statistical Computing, Vienna, Austria). The randomization list was maintained by an independent research coordinator who was not involved in treatment delivery or outcome assessment, in a password-protected electronic file accessible only to this coordinator. This same coordinator confirmed each participant’s eligibility at enrollment and subsequently disclosed the treatment assignment; treating clinicians at each site had no access to the allocation sequence prior to assignment.

Allocation was concealed until splint delivery. Because the two splints differed visibly in material, fabrication method, and chairside adjustment procedure, blinding of treating clinicians and patients after allocation was not feasible. Randomization was performed independently of clinical treatment to minimize selection bias.

### 2.4. Splint Fabrication and Delivery

**Figure 1:**
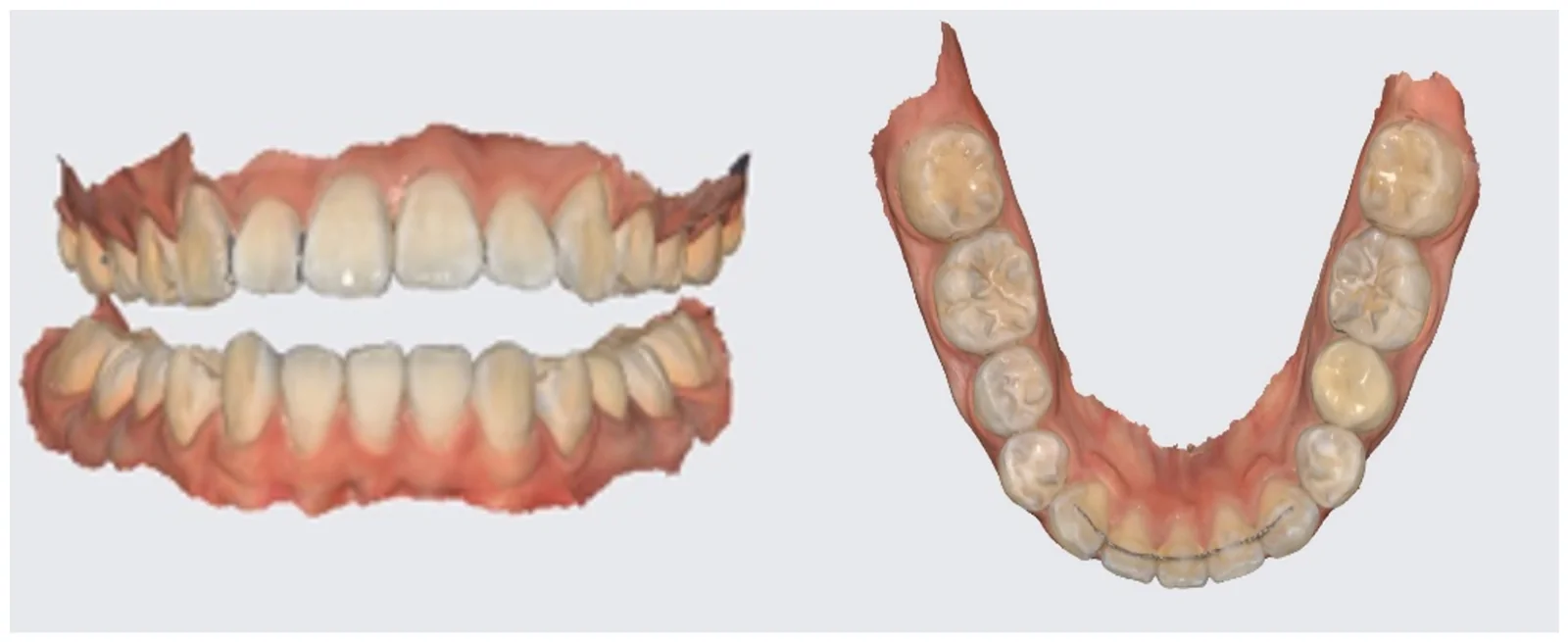
Representative stereolithography (STL) data obtained from a three-dimensional intraoral scanner (Medit i700; Medit Corp., Seoul, Republic of Korea) as part of the digital workflow for customized hard splint fabrication.

For the customized hard splint group, intraoral digital impressions were obtained using a three-dimensional intraoral scanner (Medit i700; Medit Corp., Seoul, Republic of Korea). The scanned stereolithography files were used to design customized hard stabilization splints (Figure 1).

**Figure 2:**
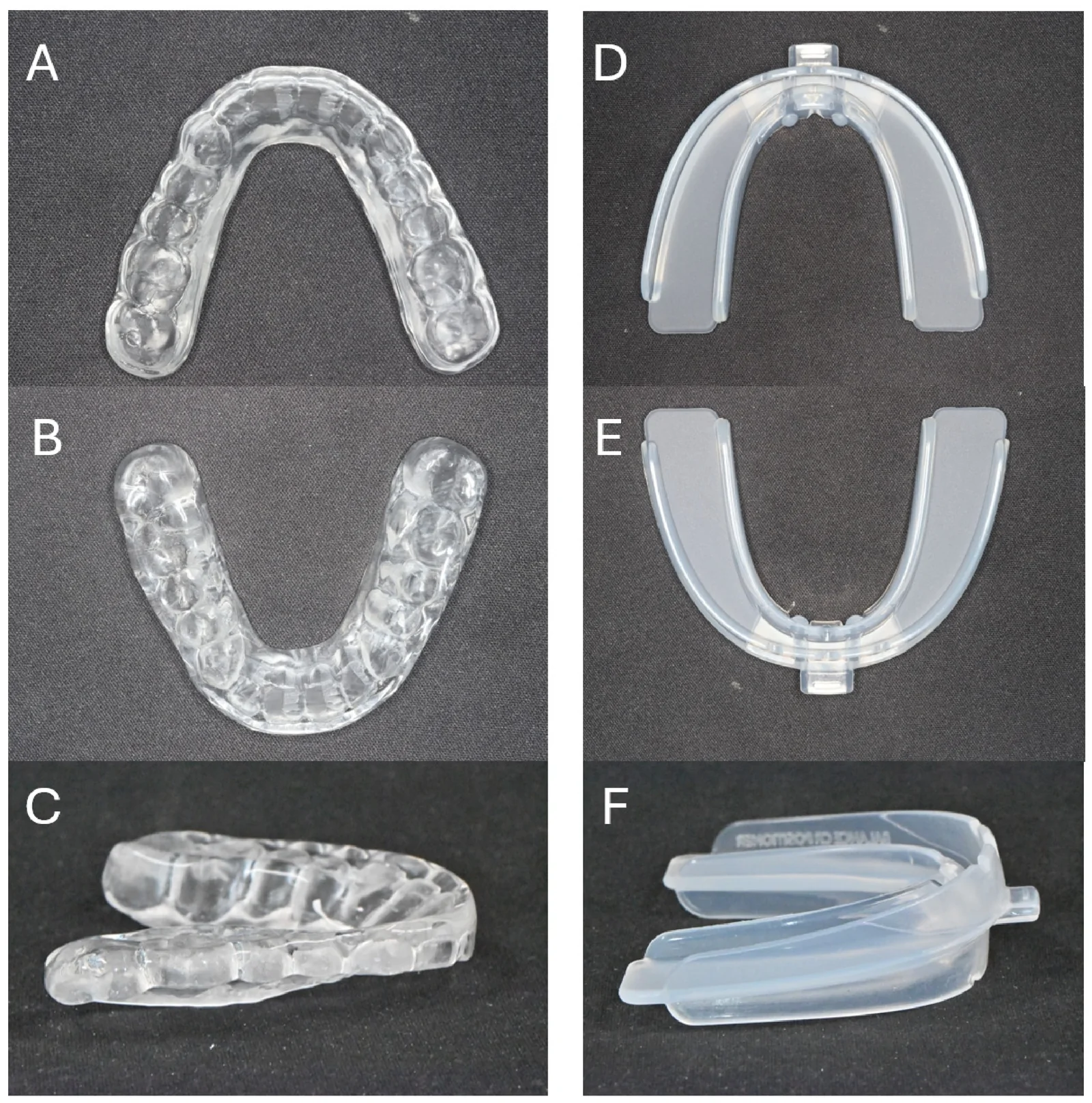
Representative splints used in this study. (**A**–**C**) Customized three-dimensional printed hard stabilization splint: (**A**) maxillary occlusal surface, (**B**) mandibular contact surface, and (**C**) sagittal aspect. (**D**–**F**) Ready-made soft splint: (**D**) maxillary occlusal surface, (**E**) mandibular contact surface, and (**F**) sagittal aspect. In both appliances, the maxillary surface covered approximately the occlusal one-third of the maxillary tooth crowns (**A**,**D**). On the mandibular side, the hard splint presented a flat occlusal contact surface, with contact points at the cusp tips of the mandibular teeth confirmed using articulating paper (**B**), whereas the soft splint’s thermoplastic material conformed more broadly to the mandibular arch, covering approximately the occlusal one-third of the mandibular tooth crowns (**E**), reflecting differences in material properties between the two appliance types rather than an intentional design difference. These features are further illustrated in the sagittal views (**C**,**F**).

Splints were fabricated from clear light-polymerizing resin (KeySplint Hard; Keystone Industries, Gibbstown, NJ, USA) using a digital light processing (DLP) 3D printer (Asiga MAX UV; Asiga, Sydney, Australia) by external dental laboratories and delivered to each participating institution. This photopolymer resin is a biocompatible, rigid splint material intended for intraoral occlusal appliance fabrication (FDA 510(k)-cleared; CE marked, Class IIa). The maxillary surface of the hard splint covered approximately the occlusal one-third of the maxillary tooth crowns, while the mandibular contact surface was flat. Following delivery, occlusal contacts were verified using articulating paper (Bausch Articulating Paper, 40 μm; Dr. Jean Bausch GmbH & Co. KG, Cologne, Germany) and adjusted to achieve simultaneous, evenly distributed bilateral contact; the resulting contact marks were localized to the cusp tips of the mandibular teeth, reflecting the natural point-contact pattern of a flat occlusal plane against cuspal tooth anatomy rather than an intentional indentation design (Figure 2A–C). At each participating institution, a single designated clinician was responsible for delivering, fitting, and adjusting both types of splints chairside. This clinician was also the one who later conducted outcome assessments at that site. To prevent bias during analysis, all outcome data were anonymized concerning group allocation before statistical evaluation. Two co-authors, who had no role in splint delivery, fitting, or adjustment and remained blinded to group assignments during data interpretation, performed the statistical analysis. No additional eligibility criteria (e.g., a specific specialty certification or a minimum number of years of clinical experience) were pre-specified for these designated clinicians beyond their routine clinical role at each site. For the ready-made soft splint group, a prefabricated transparent thermoplastic appliance (Balance CR splint; Balancetech Corp., Seongnam, Republic of Korea), registered as a medical device with the Korean Ministry of Food and Drug Safety (MFDS), was delivered chairside at the initial visit without the need for impression-taking or laboratory fabrication. The appliance was trimmed with scissors along the margins to conform to each patient’s individual arch form and to eliminate contact with the tongue, gingiva, or buccal mucosa. As with the hard splint, the maxillary surface of the soft splint covered approximately the occlusal one-third of the maxillary tooth crowns; the mandibular contact surface conformed more broadly to the occlusal one-third of the mandibular tooth crowns, reflecting the moldable, thermoplastic nature of the appliance material (Figure 2D–F). Correct seating was verified by instructing each patient to close gently onto the posterior teeth while producing a lip-closed humming sound (‘M’ sound), which facilitated natural mandibular positioning and confirmed stable posterior occlusal contact with the appliance in situ.

At the delivery visit, each splint was checked for fit, retention, patient comfort, and the absence of traumatic occlusal contact or soft-tissue irritation. Patients in both groups were instructed to wear the assigned splint for 8–10 h each night during sleep. In addition, patients in both groups were instructed to perform light chewing exercises against the appliance for approximately 1–3 h during the day, using minimal biting force. Thereafter, patients were advised to continue nighttime use and to increase wearing time when clinically indicated, except during eating and tooth brushing.

The daytime chewing exercise was intended to promote splint adaptation and masticatory muscle relaxation and reflected the treating clinicians’ clinical practice rather than a standardized published protocol. No validated published protocol combining nighttime splint wear with this specific daytime chewing exercise was used. Adherence to the prescribed nighttime wear duration and daytime chewing exercises was assessed verbally at each follow-up visit and documented in the clinical record. All patients who completed follow-up were reported to have adapted well to splint use; however, adherence was not objectively quantified using wear-time logs or sensors. Participants were also asked to refrain from other TMD-directed treatments, such as additional analgesics for TMD symptoms, physical therapy, or alternative appliances, for the duration of the study period.

### 2.5. Outcome Evaluation

At baseline, all patients underwent structured history-taking and standardized clinical examination for TMD-related symptoms, including assessment of maximum mouth opening, mandibular deviation during opening, TMJ clicking, TMJ pain, and masticatory muscle pain. Masticatory muscle pain was assessed by palpating the masseter and temporalis muscles using a standardized protocol. At each participating institution, outcome assessments were performed by a single designated examiner throughout the study period to maintain intra-examiner consistency. The two site examiners reviewed the modified Helkimo index scoring criteria together prior to study commencement to promote cross-site uniformity; however, formal inter-examiner reliability testing was not performed. Given that the outcome assessor at each site was the identical clinician responsible for delivering and adjusting the assigned splint, and considering that the two splints displayed visible differences in material that could not be concealed, this assessor was not blinded to the treatment allocation during clinical scoring. The measures implemented to mitigate potential bias during the analysis phase are detailed in Section 2.6.

Clinical outcomes were evaluated using the modified Helkimo index at baseline (T0), 4 weeks after splint delivery (T1), and 12 weeks after splint delivery (T2) (Table 1). Because fabrication of the customized hard splint required approximately one week from digital impression to delivery, the interval between baseline assessment and splint delivery differed between the two groups; however, both groups were evaluated using an identical follow-up frame anchored to each patient’s own splint delivery date, ensuring equivalent intervention exposure duration at T1 and T2 across groups. Each component was scored as 0, 1, or 5 according to the severity of the clinical finding, and the total score was calculated by summing the five component scores. The degree of dysfunction was classified as follows: 0, no dysfunction; 1–4, mild dysfunction; 5–9, moderate dysfunction; and ≥10, severe dysfunction.

The co-primary outcomes were the between-group differences in change in total modified Helkimo index score from baseline to T1 and from baseline to T2. These comparisons were exploratory and were intended to assess between-group differences in short-term clinical trajectories, not to establish non-inferiority or equivalence.

At the final follow-up visit (T2, 12 weeks), occlusal changes were assessed by the same designated examiner using visual inspection and shimstock foil (HANEL Shimstock Foil 8 μ; Coltène, Altstätten, Switzerland) occlusal contact evaluation. Any change in the distribution or presence of occlusal contacts compared with baseline was recorded. This assessment was performed to monitor for potential appliance-related occlusal side effects, particularly given the non-individualized nature of the ready-made soft splint. Safety and tolerability were also pre-specified for monitoring at each visit, encompassing appliance-related soft-tissue irritation or discomfort, the need for chairside adjustment, and appliance breakage or deformation; these findings are reported in Section 3.5.

Visual inspection combined with shimstock foil evaluation constitutes a chairside, examiner-dependent method primarily capable of detecting significant loss or gain of occlusal contact. However, it exhibits limited sensitivity to more subtle changes in contact intensity, timing, or distribution.

### 2.6. Statistical Analysis

Statistical interpretation and analysis were conducted by two co-authors who had no involvement in splint application, fitting, or adjustment at any site; group allocation was concealed in the dataset (coded as Group A/Group B) during data interpretation and statistical analysis in order to minimize bias during the analysis stage.

Changes in the total modified Helkimo index scores from baseline to 4 weeks (T0–T1) and from baseline to 12 weeks (T0–T2) were calculated for each patient. Negative change scores indicate improvement (follow-up score minus baseline score). Two participants in the ready-made soft splint group discontinued before their first post-baseline assessment, with no T1 or T2 data available for either. Because neither discontinuation was reported to involve an adverse event, treatment dissatisfaction, or protocol deviation, the missing-at-random (MAR) assumption was judged reasonable, although it could not be formally tested; the possibility that either discontinuation was related to an unrecorded aspect of the patient’s clinical status cannot be fully excluded. A complete-case analysis (n = 35 for the hard splint group, n = 33 for the soft splint group) was therefore adopted as the primary analytical approach. A separate per-protocol analysis was not conducted, since splint-wear adherence was not objectively verified.

As a sensitivity analysis, a tipping-point approach was used in place of a single best-case/worst-case imputation: the change in modified Helkimo index score from baseline to T2 for the two missing soft splint group participants was imputed across assumed values, from no improvement (change = 0) to a large improvement (changes = −7 and −6, respectively, corresponding to each participant’s maximum possible improvement), and the between-group comparison (Mann–Whitney U test) was repeated at each assumed value, together with an intention-to-treat (ITT) analysis including all 70 randomized participants using baseline-observation-carried-forward (no change assumed).

Continuous variables are presented as mean ± standard deviation; due to the modified Helkimo index summing five components, each scored 0, 1, or 5, its total score does not conform to an interval scale and may exhibit a floor effect, especially at later follow-up. Consequently, nonparametric methods were used as the primary analytic approach for all inferential comparisons, rather than a parametric model. Categorical variables are presented as number and percentages.

Within-group changes from baseline at T1 and T2 were evaluated separately for each type of splint using the Wilcoxon signed-rank test. Inter-group comparisons of (i) raw modified Helkimo index scores at T1 and T2, and (ii) individual change scores from baseline to T1 and T2, were conducted using the Mann–Whitney U test; when reported, the between-group median difference and its 95% confidence interval were estimated using the Hodges-Lehmann estimator. Both T1 and T2 were predetermined as co-primary time points, and no adjustments for multiple comparisons were applied across these two time points. Baseline (T0) scores were compared between groups using an independent-samples *t*-test.

As an additional sensitivity analysis, addressing the numerical, non-significant baseline imbalance between groups (Table 2), the differences between groups in the change of the modified Helkimo index score from baseline to T1 and from baseline to T2 were further estimated using analysis of covariance (ANCOVA). This was specified as a linear regression of the change score on splint type, baseline (T0) score, and institution. The model directly provides an adjusted between-group mean difference (customized hard splint minus ready-made soft splint), along with its 95% confidence interval and an exact *p*-value, accounting for both baseline severity and institutional site.

Statistical significance was established at *p* < 0.05. All analyses were conducted using IBM SPSS Statistics for Windows, version 22.0 (IBM Corp., Armonk, NY, USA), along with Python (version 3.11; SciPy version 1.18.1 and statsmodels version 0.15.0 libraries) for Hodges-Lehmann estimation and ANCOVA sensitivity analysis.

### 2.7. Ethics Approval

This study was conducted in accordance with the Declaration of Helsinki. The study protocol was approved by the Institutional Review Board of Hallym University Sacred Heart Hospital (IRB No. 2023-01-011; approved on 21 February 2023) and by the Public Institutional Review Board Designated by the Ministry of Health and Welfare, Republic of Korea, for Balance dental hospital (Approval No. P01-202303-01-016; approved on 18 October 2023). Written informed consent was obtained from all participants before enrollment.

### 2.8. Trial Registration

This exploratory randomized study was retrospectively registered in the Clinical Research Information Service (CRIS), Republic of Korea (Registration No. KCT0012318; registered 22 July 2026). The clinical outcome variable and the co-primary assessment time points (T1 and T2) were specified from the outset. However, a separate formal statistical analysis plan was not prepared before enrollment. The final statistical methods used in this report were selected after data collection, based on consultation with a biostatistician and subsequent peer-review considerations. Prospective registration was not completed prior to participant enrollment, which is acknowledged as an important limitation. The original IRB-approved study protocol is available from the corresponding author upon reasonable request.

Participant enrollment and follow-up were conducted from 2 March 2023 (first enrollment, Hallym University Sacred Heart Hospital) through 16 July 2024 (last follow-up visit, Balance dental hospital).

### 2.9. Generative AI Use

**Figure 3:**
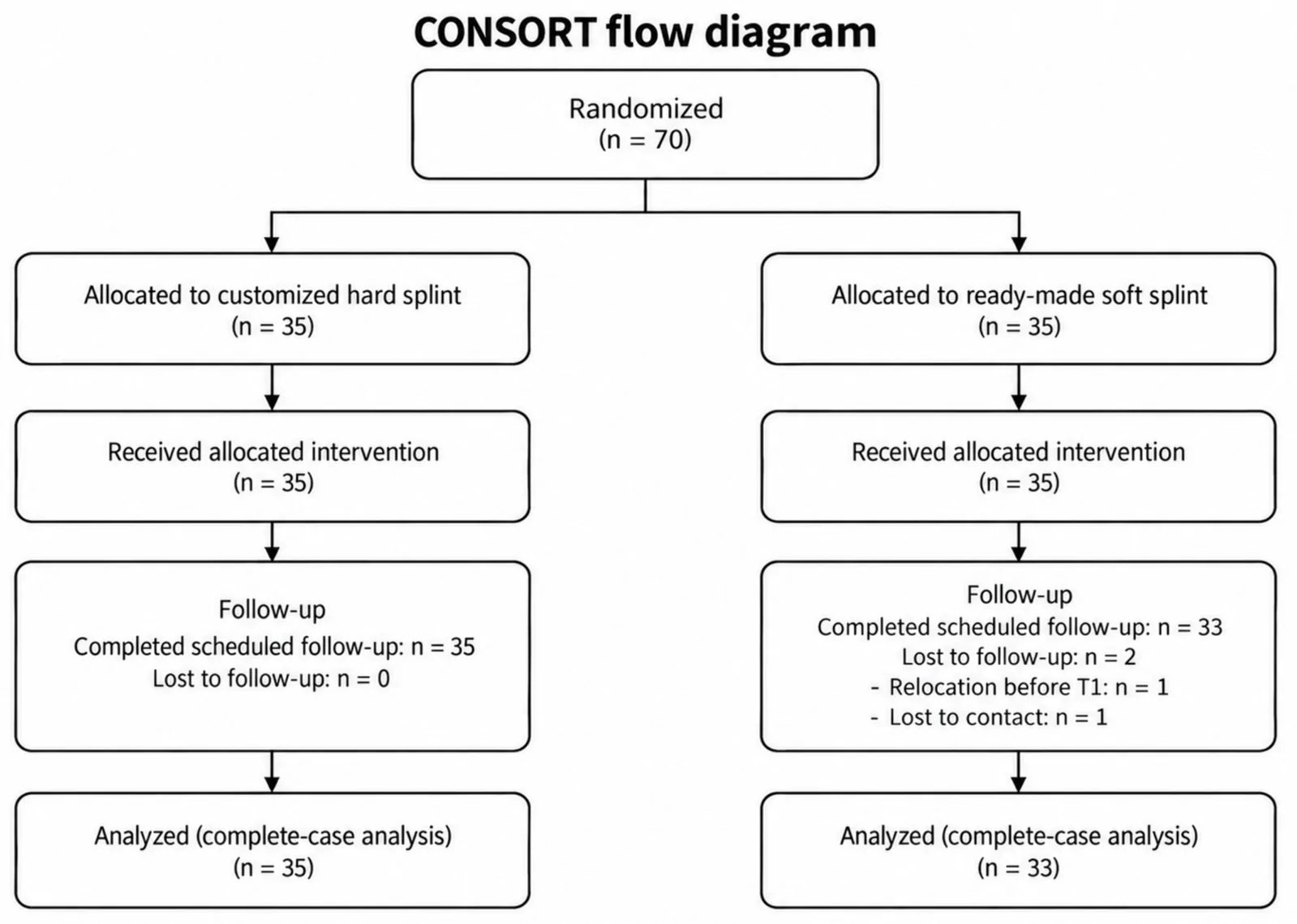
CONSORT flow diagram of patient enrollment, randomization, allocation, receipt of allocated intervention, follow-up, and analysis. A total of 70 patients were randomized to the customized hard splint group (n = 35) or the ready-made soft splint group (n = 35); all 70 patients received their allocated intervention. Two patients in the soft splint group were lost to follow-up before T1 (relocation, n = 1; lost to contact, n = 1). Neither dropout was reported to be associated with splint-related adverse effects or treatment dissatisfaction. Sixty-eight patients were included in the final complete-case analysis (customized hard splint group, n = 35; ready-made soft splint group, n = 33).

**Figure 4:**
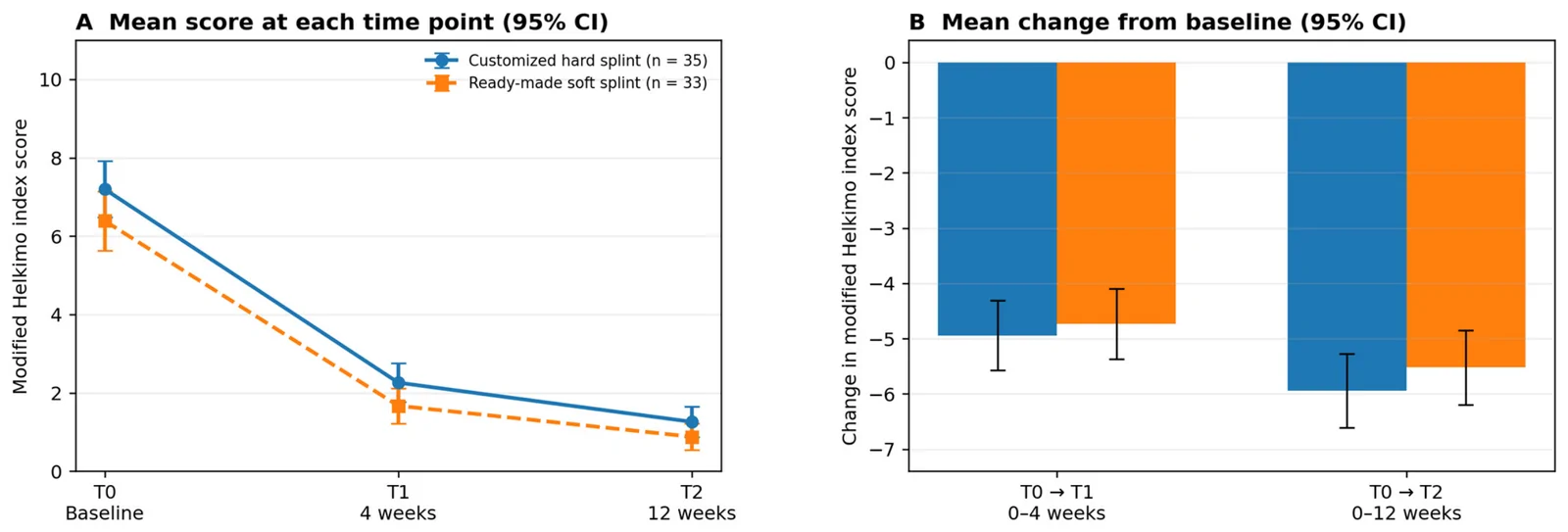
Changes in modified Helkimo index scores after splint therapy. (**A**) Mean modified Helkimo index scores with 95% confidence intervals at baseline (T0), 4 weeks (T1), and 12 weeks (T2) in the customized hard splint and ready-made soft splint groups. (**B**) Mean changes in modified Helkimo index scores from baseline to 4 weeks (T0–T1) and from baseline to 12 weeks (T0–T2). Negative values indicate improvement from baseline. Between-group comparisons were performed using the Mann–Whitney U test. ns, not significant; CI, confidence interval.

ChatGPT (GPT-5.6 Sol; OpenAI, San Francisco, CA, USA) was used to assist in the preparation of Figure 3 and Figure 4. The CONSORT flow diagram (Figure 3) was generated systematically from author-provided data regarding enrollment, allocation, follow-up, and analysis. The outcome graphs (Figure 4) were produced programmatically using Python and Matplotlib (version 3.11.2), based on the numerical results presented in Section 3.3.

## 3. Results

### 3.1. Patient Flow and Baseline Characteristics

A total of 70 patients were enrolled and randomly allocated to the customized hard splint group (n = 35) or the ready-made soft splint group (n = 35). All 35 patients in the hard splint group completed the scheduled follow-up evaluations. In the ready-made soft splint group, two patients did not complete follow-up assessments and were excluded from the complete-case analysis: one patient relocated to another city before the T1 visit, and the other was lost to follow-up despite repeated attempts. Neither case was associated with splint-related discomfort, adverse effects, or treatment dissatisfaction. Consequently, 68 patients were included in the final analysis (hard splint group, n = 35; soft splint group, n = 33). The flow of enrollment, randomization, allocation, receipt of allocated intervention, follow-up, and analysis is summarized in Figure 3.

Baseline demographic and clinical characteristics for all randomized participants are summarized in Table 2. There were no statistically significant between-group differences in age, sex, symptom duration, baseline modified Helkimo index score, or dysfunction severity.

### 3.2. Within-Group Changes in Modified Helkimo Index Scores

Both groups showed statistically significant reductions in total modified Helkimo index scores at T1 and T2 compared with baseline (all *p* < 0.001). In the customized hard splint group, the mean total score decreased from 7.20 ± 2.10 at T0 to 2.26 ± 1.44 at T1 and 1.26 ± 1.12 at T2 (Table 3). In the ready-made soft splint group (complete-case analysis, n = 33), the mean total score decreased from 6.39 ± 2.14 at T0 to 1.67 ± 1.27 at T1 and 0.88 ± 0.93 at T2 (Table 4).

### 3.3. Between-Group Comparison of Clinical Improvement

Comparative analysis of the raw modified Helkimo index scores across different groups at each specified time point is summarized in Table 5. At baseline, the mean total modified Helkimo index score was 7.20 ± 2.10 within the hard splint group and 6.39 ± 2.14 within the soft splint group (independent-samples *t*-test, *p* = 0.121). At T1, the mean scores were 2.26 ± 1.44 for the hard splint group and 1.67 ± 1.27 for the soft splint group; the Mann–Whitney U test did not reveal a statistically significant difference between the groups (Hodges-Lehmann median difference = 1.00, 95% CI 0.00 to 1.00, *p* = 0.074). At T2, the mean scores were 1.26 ± 1.12 in the hard splint group and 0.88 ± 0.93 in the soft splint group (Hodges-Lehmann median difference = 0.00, 95% CI 0.00 to 1.00, *p* = 0.160).

A comparison of the changes in the modified Helkimo index score from baseline between groups is summarized in Table 6. The Mann–Whitney U test revealed no statistically significant difference between groups in the magnitude of improvement at T0–T1 (Hodges-Lehmann median difference, hard splint minus soft splint = 0.00, 95% CI −1.00 to 1.00; *p* = 0.573) or at T0–T2 (Hodges-Lehmann median difference = −1.00, 95% CI −2.00 to 0.00; *p* = 0.209). To examine whether the outcome patterns were descriptively similar across sites, results were additionally tabulated by institution (Supplementary Table S2). The direction and approximate magnitude of within-group improvement were similar at Hallym University Sacred Heart Hospital and Balance dental hospital, with a numerically greater T0–T2 reduction in the hard splint group at each site. These site-stratified summaries were descriptive, and no site-specific inferential comparisons were performed. No obvious qualitative inconsistency in outcome patterns was observed between the two institutions. To address the numerical baseline imbalance between groups, a sensitivity analysis using ANCOVA, adjusted for baseline score and institution, was conducted for change in the modified Helkimo index score at T1 and T2 (Supplementary Table S1). The adjusted between-group mean differences remained small and statistically non-significant at T0–T1 (adjusted difference = 0.32, 95% CI −0.26 to 0.90, *p* = 0.278) and T0–T2 (adjusted difference = 0.21, 95% CI −0.26 to 0.68, *p* = 0.369), consistent with the overall inferential conclusion of the primary Mann–Whitney U analyses. The signs of these adjusted differences were opposite to those of the unadjusted mean differences in change (−0.21 at T0–T1 and −0.42 at T0–T2; Table 6).

### 3.4. Score Distribution and Floor Effect

Considering the non-interval, floor-affected nature of the modified Helkimo index total score, the distribution of scores is further summarized using medians, interquartile ranges (IQRs), and the proportion of participants achieving a score of zero (Table 7). A floor effect was observed by T2: nine out of thirty-five patients (25.7%) in the hard splint group and thirteen out of thirty-three patients (39.4%) in the soft splint group had a total score of zero, indicating an absence of detectable dysfunction according to this index. Category-level changes from baseline to T2 (Supplementary Table S3) showed that all patients who initially fell within the moderate (DiII) or severe (DiIII) dysfunction categories transitioned to lower categories by T2 in both groups.

### 3.5. Safety and Tolerability

No clinically significant adverse events, appliance breakage, deformation, or need for remake occurred in either group during the 12-week follow-up period. At each follow-up visit, patients were assessed for soft-tissue irritation and appliance-related discomfort; no clinically significant findings were identified on examination in either group, and no patient reported discomfort related to splint use at any follow-up visit. No patient temporarily discontinued splint use because of an adverse effect. Minor chairside adjustments were required in five female patients in the soft splint group with smaller oral cavities, who required scissor trimming of the appliance margins at the initial delivery visit for fit; no mid-treatment occlusal adjustments were performed in either group, as none were clinically indicated. Occlusal contact assessment at 12 weeks (T2), performed by visual inspection and shimstock foil evaluation, revealed no clinically detectable change in occlusal contacts compared with baseline in either group. Sleep disturbance, changes in pain intensity, and loss of retention were not systematically recorded as discrete, quantified adverse-event categories at either site.

### 3.6. Sensitivity Analysis: Missing Data (ITT and Tipping-Point Analysis)

An ITT analysis with all 70 participants, using baseline-observation-carried-forward for the two individuals lost to follow-up (assuming no improvement), did not significantly alter the conclusion of no between-group difference in the change of the modified Helkimo index score from baseline to T2 (*p* = 0.106). A tipping-point sensitivity analysis, which varied the assumed change score for the missing participants from no improvement to maximum improvement, likewise did not yield a significant difference at any value (*p* range, 0.106–0.261).

## 4. Discussion

The principal finding of this exploratory randomized study was that modified Helkimo index scores decreased significantly from baseline in both treatment groups at 4 and 12 weeks, with no statistically significant between-group difference in the magnitude of change. Because all participants also received standardized counseling, the within-group decreases cannot be attributed to either splint alone. Given the exploratory nature of the study, these findings should be regarded as preliminary and hypothesis-generating.

The two study arms represented distinct clinical pathways that differed simultaneously in customization, fabrication process, material properties, occlusal design, delivery workflow, and time to delivery. Accordingly, the observed outcomes cannot be attributed to any single factor such as material hardness, customization, or digital fabrication alone. Isolating these components would require a factorial or multi-arm design and was beyond the scope of the present exploratory trial.

The customized hard splint provides individualized fit and occlusal adjustment but requires digital scanning, laboratory fabrication, and a delay before delivery. In contrast, the ready-made soft splint can be adjusted and delivered chairside but lacks individualized occlusal design and may be less suited to sustained heavy parafunctional loading [10,29,30,31]. These practical differences form part of the distinct clinical pathways compared in this study and should not be interpreted as evidence that either appliance is preferable for clinical situations not directly evaluated here.

Occlusal splints are widely used as conservative, reversible treatment options for TMDs, along with patient education, behavioral modification, physical therapy, and pharmacologic management [6,7,8,9]. Their clinical use is supported by their reversibility and potential to reduce pain and improve mandibular function in appropriately selected patients [10,11,32]. Stabilization splints are generally preferred over anterior repositioning splints for routine TMD symptom management owing to their broader evidence base and lower risk of inducing permanent occlusal changes [32]. The therapeutic effects of stabilization-type appliances are likely multifactorial, involving reductions in deflective occlusal contacts, redistribution of occlusal loading, limitation of parafunctional loading, and promotion of neuromuscular relaxation [10,11,33,34]. A previous clinical study reported comparable outcomes with soft and hard stabilization splints, suggesting that short-term clinical improvement may be more closely related to functional stabilization and neuromuscular modulation than to material composition alone [10]. The present findings are consistent with this interpretation.

Prior evidence has raised important considerations regarding prefabricated resilient appliances in TMD management. Soft splints lack individualized occlusal adjustment, have in some settings increased rather than reduced masticatory muscle activity in patients with bruxism, and have ranked below hard stabilization splints for pain reduction in arthrogenous TMDs [10,29,30,31]. These concerns are well founded, and the present study does not position ready-made soft splints as a substitute for individualized hard stabilization appliances in routine clinical practice.

The findings are interpreted within a specific and limited clinical context: a supervised, short-term (12-week) intervention in a clinically heterogeneous adult TMD population, including patients with mild, moderate, and severe dysfunction and overlapping myogenous and arthrogenous features, formally characterized only as meeting DC/TMD criteria for TMD rather than by a structured diagnostic subtype. Because structured, patient-level diagnostic subtype data (myogenous vs. arthrogenous) were not retained and this study was not powered for diagnosis-specific comparisons, no phenotype-specific subgroup analysis was performed. Patients with confirmed sleep bruxism, disc displacement without reduction, or significant occlusal instability were not enrolled as a targeted subgroup, and the results should not be generalized to these populations. In particular, the management of disc displacement without reduction represents a distinct clinical challenge that may require more targeted multimodal approaches beyond splint therapy alone [35]. In this supervised, short-term setting, both appliances were associated with statistically significant short-term clinical improvement. For patients presenting with TMJ pain in the absence of radiographic evidence of osseous destruction, clinicians often seek a treatment option that can be initiated immediately; however, the fabrication delay and relatively higher cost of a customized hard splint may reduce patient adherence at this initial stage. These findings are similarly limited to the adult TMD population actually enrolled and do not extend to patients undergoing active orthodontic treatment, with growing or developing dentitions, or with diagnostic uncertainty; whether ready-made soft splints could serve a pragmatic bridging role in any of these populations, or in patients with confirmed bruxism or occlusal instability, remains untested and is a question for dedicated future study.

Although this study examined the pragmatic clinical question of whether a ready-made soft splint and a customized hard splint would show a detectable difference in short-term symptomatic improvement, it was not designed as a formal non-inferiority or equivalence trial. A pre-specified non-inferiority margin, dedicated power calculation, and confidence interval-based decision rule were not established. Therefore, the absence of between-group differences should not be taken as evidence of non-inferiority or equivalence, and it reflects only that no significant difference was detected within the limits of this exploratory study, and no claim of comparable effectiveness is made. Future non-inferiority or equivalence trials should define a clinically meaningful margin in advance and use confidence interval-based interpretation [36,37].

The customized hard splint was manufactured using a fully digital workflow incorporating intraoral scanning, virtual design, and DLP 3D printing [12,13,14]. Whether potential advantages of digital personalization, such as improved fit accuracy, translate into measurable clinical benefits over longer treatment periods or in specific patient subgroups remains uncertain. Future studies incorporating objective measures of appliance fit and material performance alongside clinical outcomes may help clarify the clinical contexts in which digital fabrication provides additional benefit.

At baseline, the mean total modified Helkimo index score was numerically higher in the hard splint group than in the soft splint group (7.20 vs. 6.40). However, this difference did not reach statistical significance (*p* = 0.113). The mean baseline modified Helkimo index score in both groups fell within the moderate dysfunction range, although individual participants included mild, moderate, and severe presentations. The between-group difference in mean baseline score was 0.80 points. Because numerical baseline imbalance may influence an unadjusted estimate of change and reduce precision, a baseline- and institution-adjusted ANCOVA sensitivity analysis was performed in the complete-case analytic population (Supplementary Table S1). The adjusted between-group differences remained small and statistically non-significant at both T1 and T2; however, because adjustment changed the direction and magnitude of the estimated contrasts, an influence of the baseline imbalance on the treatment estimates cannot be excluded. Future studies should nonetheless consider stratified randomization based on baseline dysfunction severity to minimize this potential source of bias at the design stage.

This study did not include an untreated, counseling-only, or sham-appliance comparison group. All participants received standardized counseling on TMD education and parafunctional habit avoidance, in addition to their assigned splint. Therefore, the clinical assessments cannot distinguish the splint’s specific contribution from that of the counseling, the natural waxing-and-waning course of TMD symptoms, regression to the mean following enrollment during a period of symptom exacerbation, or nonspecific effects of trial participation and regular clinician contact. Consequently, the within-group score reductions observed in both arms should not be interpreted as evidence that either splint, in isolation, caused the observed clinical improvement; this trial can speak only to the relative short-term trajectory of the two appliance-plus-counseling regimens compared with each other, not to their effect relative to no treatment. Future trials incorporating a no-treatment, waitlist, or counseling-only control arm would be needed to isolate the specific contribution of splint therapy.

The registration of the trial was not conducted prospectively; instead, the study was registered retrospectively in CRIS. This is recognized as a significant methodological limitation, as is the lack of a pre-specified non-inferiority or equivalence design [37]. Furthermore, because no multiplicity adjustment was applied to the two co-primary time-point comparisons, the family-wise type I error rate was not formally controlled. Accordingly, the findings at T1 and T2 should be interpreted as exploratory and hypothesis-generating rather than confirmatory.

Blinding of patients and treating clinicians after allocation was not feasible given the visible differences between the two appliances. The outcome assessor at each site was the same clinician who delivered and adjusted the assigned splint, and was therefore also not blinded to treatment allocation at the time of scoring; combined with the site-level conflict of interest, this is a genuine risk of detection bias. Raw outcome data were subsequently masked to group assignment and independently analyzed by two co-authors not involved in splint delivery at either site, but this blinded statistical interpretation does not substitute for blinded outcome assessment, and in the absence of formal inter-examiner reliability testing, no quantitative measure such as Cohen’s kappa was obtained; the possibility that unblinded, site-specific scoring influenced the results cannot be entirely excluded; the institution-adjusted ANCOVA (Supplementary Table S1) and site-stratified results (Supplementary Table S2) offer some reassurance but cannot substitute for a formal reliability assessment.

Two patients in the soft splint group were lost to follow-up before T1 (relocation, n = 1; lost to contact, n = 1). Neither dropout was reported to be associated with splint-related adverse effects or treatment dissatisfaction, and intention-to-treat and tipping-point sensitivity analyses did not change the overall inferential conclusion.

Splint adherence, including the daytime chewing exercise (a practice-based component not tied to a single validated protocol), was assessed via unverified patient self-report rather than objective measurement.

Because structured, patient-level DC/TMD Axis I diagnostic subtyping and Axis II psychosocial assessment were not performed, the diagnostic composition of this heterogeneous sample and its potential influence on treatment response could not be evaluated. Consequently, the findings should not be presumed to be universally applicable across TMD subtypes. Future studies are recommended to implement the comprehensive DC/TMD Axis I/II protocol, including pre-specified subgroup analyses.

The follow-up period was limited to 12 weeks. Since the ready-made splint is not customized to the patient’s occlusion, extended use involves a theoretical risk of unintended occlusal changes; however, none were observed within the 12-week duration of this study. Consequently, findings should not be generalized to long-term application. Occlusal alterations were evaluated solely through visual inspection and shimstock foil, a chairside method that lacks sensitivity to subtle variations in contact intensity, timing, or distribution. More objective assessment techniques, such as T-scan, were not employed. Additionally, minor tolerability categories, including sleep disturbance, changes in pain intensity, and loss of retention, were not systematically documented, thereby limiting the ability to formally compare tolerability between the appliances.

The modified Helkimo index sums five components scored 0, 1, or 5, producing an uneven-interval score with a hard floor. By T2, roughly one-quarter to two-fifths of participants in each group scored zero (Table 7), limiting further detectable improvement. No validated minimal clinically important difference (MCID) has been established for this index. Therefore, the non-significant *p*-values reported here should not be interpreted as indicating whether the observed differences are, or are not, clinically meaningful. Component-level scores were not retained separately, precluding component-level analysis. This study also did not include patient-reported pain, jaw functional limitation, quality-of-life, or psychosocial measures; future studies should incorporate such multidimensional outcomes, prospective registration, and longer follow-up.

Modified Helkimo index scores decreased significantly in both groups over the 12-week period, with no significant between-group difference detected. These findings raise the hypothesis that ready-made soft splints may have a limited short-term role in selected clinical situations where customized fabrication is impractical or delayed; this possibility requires confirmation in appropriately designed studies.

## 5. Conclusions

In this exploratory randomized study, modified Helkimo index scores decreased significantly from baseline in both treatment groups, with no statistically significant between-group difference in change detected over the 12-week follow-up period. This absence of a detected difference should not be interpreted as evidence of equivalence or non-inferiority. These findings are preliminary and hypothesis-generating; adequately powered, prospectively registered non-inferiority or equivalence trials with longer follow-up and patient-reported outcomes are needed to define the clinical role and optimal indications of ready-made soft splints in TMD management. Given the theoretical risk of unmonitored occlusal change with prolonged use of a non-individualized appliance, use of the ready-made soft splint should not extend beyond the 12-week period evaluated in this study without re-assessment, and any such short-term use warrants appropriate patient selection and continued monitoring pending confirmatory long-term evidence.

## Figures and Tables

**Table 1:** Components and scoring criteria of the modified Helkimo index.

Component	Grade 0 (Score 0)	Grade I (Score 1)	Grade II (Score 5)
Maximum mouth opening	≥40 mm	30–39 mm	<30 mm
Mandibular deviation during opening	<2 mm	2–5 mm	>5 mm
TMJ clicking	Absent	Palpable only	Audible/evident
TMJ pain on palpation	Absent	Mild (pain on palpation)	Marked (pain response or withdrawal)
Masticatory muscle pain on palpation	Absent	Mild (pain on palpation)	Marked (pain response or withdrawal)

TMJ, temporomandibular joint.

**Table 2:** Baseline demographic and clinical characteristics.

Variable	Customized Hard Splint Group (n = 35)	Ready-Made Soft Splint Group (n = 35)	*p*-Value
Age, years	35.4 ± 12.1	34.8 ± 11.3	0.831
Sex, male/female	9/26	8/27	0.780
Symptom duration, months	5.8 ± 3.6	5.5 ± 3.3	0.731
Baseline modified Helkimo index score	7.20 ± 2.10	6.40 ± 2.08	0.113
Dysfunction severity, n (%)—Mild (score 1–4)	6 (17.1)	7 (20.0)	0.825
Dysfunction severity—Moderate (score 5–9)	21 (60.0)	22 (62.9)	
Dysfunction severity—Severe (score ≥ 10)	8 (22.9)	6 (17.1)	

Values are presented for all randomized participants (customized hard splint group, n = 35; ready-made soft splint group, n = 35) and are expressed as mean ± standard deviation or number (%), as appropriate. *p*-values were calculated using independent-samples *t*-tests for continuous variables and Pearson’s chi-square tests for categorical variables, as appropriate. The *p*-value for dysfunction severity represents the overall comparison across the mild, moderate, and severe categories.

**Table 3:** Within-group changes in modified Helkimo index scores: customized hard splint group (n = 35).

Time Point	n	Mean ± SD	95% CI	*p*-Value vs. T0
T0	35	7.20 ± 2.10	6.48–7.92	-
T1	35	2.26 ± 1.44	1.76–2.75	<0.001
T2	35	1.26 ± 1.12	0.87–1.64	<0.001

CI, confidence interval; SD, standard deviation. *p*-values indicate comparison with baseline (T0) using the Wilcoxon signed-rank test. “-” indicates the reference time point.

**Table 4:** Within-group changes in modified Helkimo index scores: ready-made soft splint group (n = 33).

Time Point	n	Mean ± SD	95% CI	*p*-Value vs. T0
T0	33	6.39 ± 2.14	5.64–7.15	-
T1	33	1.67 ± 1.27	1.22–2.12	<0.001
T2	33	0.88 ± 0.93	0.55–1.21	<0.001

CI, confidence interval; SD, standard deviation. *p*-values indicate comparison with baseline (T0) using the Wilcoxon signed-rank test. “-” indicates the reference time point.

**Table 5:** Between-group comparison of modified Helkimo index scores at each time point.

Time Point	Group	n	Mean ± SD	95% CI	Median Difference (95% CI)	*p*-Value
T0	Hard splint	35	7.20 ± 2.10	6.48–7.92	—	0.121 ^a^
	Soft splint	33	6.39 ± 2.14	5.64–7.15		
T1	Hard splint	35	2.26 ± 1.44	1.76–2.75	1.00 (0.00 to 1.00)	0.074
	Soft splint	33	1.67 ± 1.27	1.22–2.12		
T2	Hard splint	35	1.26 ± 1.12	0.87–1.64	0.00 (0.00 to 1.00)	0.160
	Soft splint	33	0.88 ± 0.93	0.55–1.21		

CI, confidence interval; SD, standard deviation. Median difference (hard splint minus soft splint) and its 95% CI were estimated using the Hodges-Lehmann estimator; *p*-values at T1 and T2 are from the Mann–Whitney U test comparing raw scores between groups. These comparisons are exploratory and were not adjusted for multiple comparisons. ^a^ The T0 *p*-value is from an independent-samples *t*-test comparing baseline scores between groups. At T0, values in this table refer to the complete-case analytic population used for the longitudinal outcome comparisons; randomized baseline characteristics for all 70 participants are presented separately in Table 2.

**Table 6:** Changes in modified Helkimo index scores from baseline.

Comparison	Group	n	Mean ± SD	95% CI	Median Difference (95% CI); *p*-Value Between Groups
T0–T1	Hard splint	35	−4.94 ± 1.85	−5.58 to −4.31	0.00 (−1.00 to 1.00); *p* = 0.573
	Soft splint	33	−4.73 ± 1.79	−5.36 to −4.09	
T0–T2	Hard splint	35	−5.94 ± 1.95	−6.61 to −5.27	−1.00 (−2.00 to 0.00); *p* = 0.209
	Soft splint	33	−5.52 ± 1.91	−6.19 to −4.84	

CI, confidence interval; SD, standard deviation; T0, baseline; T1, 4 weeks; T2, 12 weeks. Negative values indicate improvement from baseline. The final column reports the Hodges-Lehmann median difference (hard splint minus soft splint) with its 95% CI and the corresponding *p*-value from the Mann–Whitney U test. These comparisons are exploratory and were not adjusted for multiple comparisons. A baseline- and institution-adjusted mean difference with 95% CI for the same comparisons, estimated using ANCOVA, is presented in Supplementary Table S1.

**Table 7:** Distribution of modified Helkimo index scores at each time point.

Time	Group	n	Median (IQR)	n (%) Score = 0	Di0	DiI	DiII	DiIII
T0	Hard	35	7.0 (6.0–9.0)	0 (0%)	0	6	21	8
T0	Soft	35	6.0 (5.0–7.0)	0 (0%)	0	7	22	6
T1	Hard	35	2.0 (1.5–3.0)	6 (17.1%)	6	28	1	0
T1	Soft	33	1.0 (1.0–2.0)	6 (18.2%)	6	27	0	0
T2	Hard	35	1.0 (0.5–2.0)	9 (25.7%)	9	26	0	0
T2	Soft	33	1.0 (0.0–1.0)	13 (39.4%)	13	20	0	0

IQR, interquartile range. Di0, no dysfunction (score = 0); DiI, mild dysfunction (score 1–4); DiII, moderate dysfunction (score 5–9); DiIII, severe dysfunction (score ≥ 10).

## Data Availability

The original contributions presented in this study are included in the article/Supplementary Material. Further inquiries can be directed to the corresponding author.

## Supplementary Materials

The following supporting information can be downloaded at: https://www.mdpi.com/article/10.3390/biomedicines14102299/s1, Supplementary Table S1: Baseline- and institution-adjusted between-group differences in change from baseline (ANCOVA sensitivity analysis); Supplementary Table S2: Modified Helkimo index scores by institution and treatment group; Supplementary Table S3: Category-level change in Helkimo dysfunction grade from baseline (T0) to 12 weeks (T2).

## Abbreviations

TMD	temporomandibular disorder
TMJ	temporomandibular joint
DC/TMD	Diagnostic Criteria for Temporomandibular Disorders
CI	confidence interval
SD	standard deviation
CRIS	Clinical Research Information Service
MFDS	Ministry of Food and Drug Safety
CONSORT	Consolidated Standards of Reporting Trials
DLP	digital light processing
STL	stereolithography
IQRs	interquartile ranges
ANCOVA	Analysis of Covariance
ITT	intention-to-treat
