# Supplementary material for: Short-Term Clinical Outcomes of Ready-Made Soft Splints Versus Digitally Fabricated, Customized Hard Stabilization Splints in Temporomandibular Disorders: An Exploratory Randomized Study
Source: Biomedicines. 2026 Oct 10;14(10):2299. doi: 10.3390/biomedicines14102299 (PMC13702428; doi:10.3390/biomedicines14102299)
Supplement: Supplementary file 1 [file biomedicines-14-02299-s001.zip › biomedicines-4564981-supplementary.pdf]

Supplementary Materials

Supplementary Table S1. Baseline- and institution-adjusted between-group differences in change from baseline (ANCOVA sensitivity analysis).

Comparison	Adjusted mean, hard splint	Adjusted mean, soft splint	Adjusted mean difference (hard − soft)	95% CI	p-value
T0–T1 change	−4.68	−5.00	0.32	−0.26 to 0.90	0.278
T0–T2 change	−5.63	−5.84	0.21	−0.26 to 0.68	0.369

CI, confidence interval. Adjusted means and the adjusted mean difference were estimated using ANCOVA (linear regression of the change score on splint type, baseline T0 score, and institution). Positive values indicate a numerically smaller (less favorable) improvement in the hard splint group relative to the soft splint group. The analysis included participants with complete follow-up (hard splint group, n = 35; soft splint group, n = 33).

Supplementary Table S2. Modified Helkimo index scores by institution and treatment group.

Institution	Group	n	T0, mean ± SD	T1, mean ± SD	T2, mean ± SD	Change T0–T2, mean ± SD
Hallym	Hard	20	7.40 ± 2.37	2.20 ± 1.64	1.50 ± 1.15	−5.90 ± 2.43
Hallym	Soft	18	6.33 ± 2.06	1.83 ± 1.29	0.83 ± 0.79	−5.50 ± 2.07
Balance	Hard	15	6.93 ± 1.71	2.33 ± 1.18	0.93 ± 1.03	−6.00 ± 1.13
Balance	Soft	15	6.47 ± 2.29	1.47 ± 1.25	0.93 ± 1.10	−5.53 ± 1.77

SD, standard deviation. Values reflect patients with complete follow-up at each site (soft splint group, n = 33 total at T1/T2 across both sites, reflecting the two dropouts).

Supplementary Table S3. Category-level change in Helkimo dysfunction grade from baseline (T0) to 12 weeks (T2).

Group	Transition (T0 → T2)	n
Hard splint	DiI → Di0	3
Hard splint	DiI → DiI	3
Hard splint	DiII → Di0	6
Hard splint	DiII → DiI	15
Hard splint	DiIII → DiI	8
Soft splint	DiI → Di0	5

Soft splint	DiI → DiI	2
Soft splint	DiII → Di0	7
Soft splint	DiII → DiI	13
Soft splint	DiIII → Di0	1
Soft splint	DiIII → DiI	5

Di0, no dysfunction; DiI, mild dysfunction; DiII, moderate dysfunction; DiIII, severe dysfunction. All patients with complete follow-up showed a stable or improved dysfunction category from baseline to T2. Transition analyses include only participants with complete T0 and T2 data (hard splint group, n = 35; soft splint group, n = 33). Accordingly, the soft-splint baseline category totals in this table exclude the two participants lost to follow-up and therefore differ from the full randomized T0 distribution shown in Table 7.
